# Correlating carbon and oxygen isotope events in early to middle Miocene shallow marine carbonates in the Mediterranean region using orbitally tuned chemostratigraphy and lithostratigraphy

**DOI:** 10.1002/2014PA002716

**Published:** 2015-04-13

**Authors:** Gerald Auer, Werner E. Piller, Markus Reuter, Mathias Harzhauser

**Affiliations:** ^1^ Institute for Earth SciencesUniversity of Graz, NAWI GrazGrazAustria; ^2^ Geological‐Paleontological DepartmentNatural History Museum ViennaViennaAustria

**Keywords:** orbital tuning, event stratigraphy, Monterey Excursion, gamma ray, magnetic susceptibility, early to middle Miocene

## Abstract

During the Miocene prominent oxygen isotope events (Mi‐events) reflect major changes in glaciation, while carbonate isotope maxima (CM‐events) reflect changes in organic carbon burial, particularly during the Monterey carbon isotope excursion. However, despite their importance to the global climate history they have never been recorded in shallow marine carbonate successions. The Decontra section on the Maiella Platform (central Apennines, Italy), however, allows to resolve them for the first time in such a setting during the early to middle Miocene. The present study improves the stratigraphic resolution of parts of the Decontra section via orbital tuning of high‐resolution gamma ray (GR) and magnetic susceptibility data to the 405 kyr eccentricity metronome. The tuning allows, within the established biostratigraphic, sequence stratigraphic, and isotope stratigraphic frameworks, a precise correlation of the Decontra section with pelagic records of the Mediterranean region, as well as the global paleoclimatic record and the global sea level curve. Spectral series analyses of GR data further indicate that the 405 kyr orbital cycle is particularly well preserved during the Monterey Event. Since GR is a direct proxy for authigenic uranium precipitation during increased burial of organic carbon in the Decontra section, it follows the same long‐term orbital pacing as observed in the carbon isotope records. The 405 kyr GR beat is thus correlated with the carbon isotope maxima observed during the Monterey Event. Finally, the Mi‐events can now be recognized in the δ^18^O record and coincide with plankton‐rich, siliceous, or phosphatic horizons in the lithology of the section.

## Key Points

- Orbitally tuned shallow marine carbonate succession in the Mediterranean region
- Correlation of Mi‐ and CM‐events within Miocene shallow marine carbonates
- Gamma ray and magnetic susceptibility as proxies on isolated carbonate ramps

## 1 Introduction

The Oligocene and Miocene are critical time intervals in the Cenozoic climate evolution, where major climatic changes took place. All of which are well recorded in pelagic successions, especially by stable carbon and oxygen isotope records and the resulting global δ^13^C and δ^18^O stacks [e.g., *Woodruff and Savin*, 1989, 1991; *Miller et al*., 1991a; *Flower and Kennett*, 1994; *Zachos et al*., 2001b; *Westerhold et al*., 2005; *Holbourn et al*., 2007; *Mourik et al*., 2010, 2011; *John et al*., 2011]. Particularly, the middle Miocene climate optimum (MMCO) [e.g., *Zachos et al*., 2001b] representing the Neogene temperature high, followed by the middle Miocene climate transition (MMCT), embodies a major climatic shift during the Neogene, marking the beginning of the gradual transition from the MMCO into the icehouse climate of the Pleistocene [e.g., *Miller et al*., 1987; *Flower and Kennett*, 1994; *Zachos et al*., 2001b, 2008; *Billups et al*., 2004; *Holbourn et al*., 2007].

Extensive records of both the Miocene oxygen isotope excursions (Mi‐events) [*Miller et al*., 1991a], as an expression of Antarctic ice sheet dynamics, as well as the carbon isotope maxima (CM‐events) of increased organic carbon burial during the Monterey carbon isotope excursion [*Woodruff and Savin*, 1991] exist in open marine and shelf settings [e.g., *Woodruff and Savin*, 1989; *Miller et al*., 1991a; *Westerhold et al*., 2005; *Holbourn et al*., 2007; *John et al*., 2011; *Mourik et al*., 2010]. Despite their obvious impact on Earth's climate these characteristic climatic events are virtually unrecognized in shallow marine carbonates. This lack of evidence is caused by their generally poor stratigraphic resolution [*Mutti et al*., 2010]. Nevertheless, shallow marine carbonates do retain a considerable potential as climatic recorders [e.g., *Mutti et al*., 1997, 2010; *Mutti and Bernoulli*, 2003; *Brandano et al*., 2010; *Reuter et al*., 2013].

For the correlation of shallow marine carbonate records with pelagic reference sections different methods have been developed and applied more or less successfully. Biostratigraphy is still the preferred method to place shallow marine sections into the global chronostratigraphy. Since planktonic marker species are mostly rare in these settings, biostratigraphy is often of only limited use for correlation with the global chronostratigraphy [e.g., *Mutti et al*., 2010]. For this reason stable isotope stratigraphy became a favored method for the stratigraphic correlation of such sections, in order to correlate their carbonate facies to the established Neogene climate record [e.g., *Mutti et al*., 2010]. However, biostratigraphy is still necessary to prevent miscorrelations. More recent works [e.g., *Reuter et al*., 2013] have further stressed the importance of an integrated approach especially including all available biostratigraphic data for the construction of meaningful age models.

Carbon isotope stratigraphy is a widely used method for stratigraphic correlations and the paleoclimatic interpretation and timing of environmental changes [*Mutti et al*., 1997, 2006, 2010; *John et al*., 2003; *Brandano et al*., 2010; *Iryu et al*., 2010; *Reuter et al*., 2013]. However, the temporal resolution of δ^13^C stratigraphy is still comparably low, limiting environmental interpretation as well as the accuracy of the correlation of shallow marine sections to higher resolution global records. One possible way to achieve an increase in stratigraphic resolution would be the application of astrochronology and orbital tuning in order to refine stratigraphic models established with conventional methods.

Astrochronology became a standard method for the temporal refinement of the geological record [*Hilgen et al*., 2003, 2006, 2014; *Westerhold et al*., 2005; *Lirer et al*., 2009; *Mourik et al*., 2011; *Hinnov and Hilgen*, 2012; *Zeeden et al*., 2013]. Especially in the Neogene this approach led to a considerable improvement of the temporal resolution on a millennial scale [*Hilgen et al*., 2003; *Abels et al*., 2005; *Westerhold et al*., 2005; *Raffi et al*., 2006; *Lirer et al*., 2009; *Mourik et al*., 2010, 2011; *Zeeden et al*., 2013]. This high temporal resolution, however, is only available for pelagic sections, which have consistently proven to be superior records for both stratigraphic correlations, as well as climatological and/or paleoecological reconstructions [*Zachos et al*., 2001a; *Wade and Pälike*, 2004; *Pälike et al*., 2006; *Holbourn et al*., 2007].

In this study we apply astrochronological principles to two geophysical proxy records (gamma ray and magnetic susceptibility) of an already biostratigraphically and chemostratigraphically dated succession of shallow water carbonates of the Maiella Platform (Apennines, Central Italy), in order to investigate a possible expression of Mi‐ and CM‐events in the section. Furthermore, the use of orbital tuning of the section using the 405 kyr eccentricity “metronome” improved the already established age model.

## 2 Setting and Stratigraphic Model of the Decontra Section

**Figure 1:**
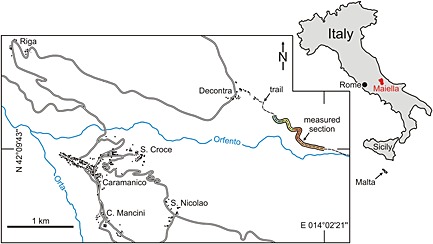
Map showing the geographical location of the Maiella mountain range in Central Italy, as well as the position of the section (highlighted area) of the mountain trail near the village of Decontra that corresponds to the logged Decontra section, with colors corresponding to the lithostratigraphic units shown in Figure 3 [after *Reuter et al*., 2013].

The studied section is located in the northwest of the Maiella mountain range in eastern Central Italy. The 120 m thick Decontra section is exposed along a trail near the village Decontra leading into the Orfento river‐valley. The base of the lithostratigraphic section is located at the GPS coordinates 42°09′43.5″N, 014°02′21.6″E at the far end of the trail from the village (Figure 1).

**Figure 2:**
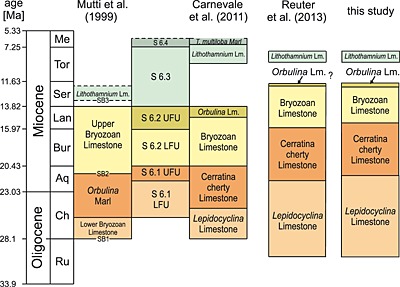
Overview of the differing stratigraphic correlation of the informal lithological units of the Decontra section after *Mutti et al*. [1997], *Vecsei and Sanders* [1999], *Carnevale et al*. [2011], and *Reuter et al*. [2013], as well as the adjustments to the stratigraphic model that resulted from the present study.

The studied Oligo‐Miocene Bolognano Formation represents the upper sedimentary succession of the Maiella Platform [*Mutti et al*., 1997; *Vecsei et al*., 1998; *Vecsei and Sanders*, 1999; *Carnevale et al*., 2011; *Brandano et al*., 2012; *Reuter et al*., 2013]. Its depositional environment was as a slightly inclined ramp on the northernmost fringe of the Apulian Platform. The Bolognano Formation can be internally subdivided into three different sequences with each being a deepening upward sequence. The sequences are further subdivided into several informal lithostratigraphic units [*Mutti et al*., 1997; *Vecsei and Sanders*, 1999; *Carnevale et al*., 2011; *Brandano et al*., 2012; *Reuter et al*., 2013]. Within the Decontra section five lithostratigraphic units are described (Figure 2):

1. *Lepidocyclina* Limestone: a 32 m thick unit composed of bioclastic grain to packstones dominated by *Lepidocyclina* and other larger benthic foraminifers. *Nephrolepidina praemarginata* occurs in the lower part and is replaced by *Nephrolepidina morgani* in the upper part.
2. Cerratina cherty Limestone: a 35 m thick succession of horizontally bedded fine bioclastic planktonic foraminiferal grainstones to packstones, containing radiolarians and siliceous sponge spicules, with intercalated layers of calcareous marls. The first occurrence of *Praeorbulina* sp. is recorded in the upper part of this unit. Phosphatization of foraminiferal tests is common.
3. Bryozoan Limestone: a 32 m thick low‐angle planar cross‐bedded grainstone dominated by bryozoan and echinoderm fragments, and planktonic and benthic foraminifers in variable quantities. Planktonic foraminifera‐dominated Limestones occur at 72 m, and between 80 and 83 m. Phosphatization is again common.
4. *Orbulina* Limestone: 3 m thick plankton rich horizon atop the Bryozoan Limestone, with abundant *Orbulina*.
5. *Lithothamnium* Limestone: 20 m thick unit of thick bedded packstones, rudstones, and floatstones, dominated by branched corallinacean debris and rhodoliths, with larger benthic foraminifers and invertebrate shells and shell fragments. The base of the *Lithothamnium* Limestone is represented by 1.5 m thick horizon of *Heterostegina* bioclastic grainstone, with a sharp contact surface to the underlying *Orbulina* Limestone. This surface is considered to represent an interruption in sedimentation [*Mutti et al*., 1997; *Carnevale et al*., 2011; *Reuter et al*., 2013].

*Reuter et al*. [2013] further revised both the lithostratigraphic characterization as well as the chemostratigraphic position of the units. Using detailed sedimentological studies, biostratigraphy, and stable carbon isotope stratigraphy, the authors were able to considerably improve the stratigraphic resolution of the Decontra section (Figure 2). Their approach using integrated proxy records largely resolved ambiguities with the stratigraphic placement of the *Lepidocyclina* Limestone, the Cerratina cherty Limestone, and the Bryozoan Limestone [*Mutti et al*., 1997; *Carnevale et al*., 2011]. Biostratigraphic ages were obtained from the *Lepidocyclina* Limestone [*Benedetti et al*., 2010; *Reuter et al*., 2013], the uppermost Cerratina cherty Limestone [*Reuter et al*., 2013], and the *Lithothamnium* Limestone [*Carnevale et al*., 2011].

## 3 Materials and Methods

During the logging of the section high‐resolution geophysical measurements, including total gamma radiation (GR) and magnetic susceptibility (MS), were carried out on site [*Reuter et al*., 2013], using a portable “GS‐512” gamma ray spectrometer (SatisGeo; measuring time 20 s) and a handheld “SM‐20” magnetic susceptibility meter (GF Instruments; sensitivity: 10^−6^ SI units). Results are reported in total counts (GR) and dimensionless SI units (MS). Measuring distances were 10 cm for GR and 5 cm for MS, based on size restriction of the used devices. Results of the measurements are summarized in Table 1 and in *Reuter et al*. [2013].

**Table 1:** Summary of the Relevant Data Sets of the Two Lithologies

	Min	Max	Average	Median	Standard Deviation
*Cerratina Cherty Limestone*					
Gamma ray	2.40	9.40	5.17	5.30	1.32
Magnetic susceptibility	0.00	91.00	13.39	11.00	12.98
Calcium carbonate	67.85%	95.64%	87.50%	88.79%	8.54 pp
TOC	0.16%	0.23%	0.19%	0.20%	0.03 pp
δ^13^C	−0.25‰	1.65‰	0.32‰	0.32‰	0.59‰
δ^18^O	−1.69‰	−0.83‰	−1.18‰	−1.17‰	0.28‰
*Bryozoan Limestone*					
Gamma ray	1.80	7.80	3.50	3.40	0.85
Magnetic susceptibility	0.00	83.00	10.10	7.00	10.43
Calcium carbonate	92.05%	98.12%	94.57%	94.37%	1.36 pp
TOC	0.07%	0.17%	0.10%	0.10%	0.02 pp
δ^13^C	0.54‰	1.78‰	1.34‰	1.46‰	0.35‰
δ^18^O	−3.47‰	2.40‰	−1.01‰	−1.09‰	1.10‰

Total organic carbon (TOC), carbonate content, and stable isotopes (δ^18^O and δ^13^C) were measured on 89 bulk samples. TOC and carbonate were measured using a LECO CS300 analyzer at the University of Graz. For TOC 0.1–0.15 g of powdered bulk sample was decalcified using 2N HCl (equaling 2M HCl or 7.3% HCl/L) prior to the LECO analysis. To calculate the carbonate content of the samples the total carbon (TC) was first measured for each bulk sample. Afterward total inorganic carbon (TIC) was calculated by subtracting the TOC from the TC content in each sample. Carbonate content was then calculated as calcite equivalent percentages using TIC with the stoichiometric formula (TIC × 8.34) [*Reuter et al*., 2013]. Results are summarized in Table 1.

For stable isotope analysis the powdered samples were dried and reacted with 100% H_3_PO_4_ at 70°C before being analyzed in an automated Kiel II preparation line and a Finnigan MAT Delta Plus mass spectrometer at the University of Graz. Repeated measurement of international standards NBS‐18 and NBS‐19 indicate an analytical precision of 0.08‰ and 0.04‰ for δ^18^O and δ^13^C, respectively. Results were normalized to the Vienna Pee Dee Belemnite standard and are reported as permil in standard delta notation. Extreme values were repeatedly measured to rule out measurement errors. Measurements are summarized in Table 1 and were discussed in *Reuter et al*. [2013] and replicate the δ^13^C measurements of *Mutti et al*. [1997].

Additionally, the mineral content of a calcareous marl occurring at ~52 m [e.g., *Reuter et al*., 2013] and another sample from the Bryozoan Limestone was determined using a Siemens D5000 X‐ray diffractometer, to resolve ambiguities concerning their classification as either siliceous Limestones or marls.

For spectral analysis only the Cerratina cherty Limestone and the Bryozoan Limestone were selected for analyses. These units showed widely continuous sedimentation and good outcrop conditions, which allowed both lithological units to be measured without significant gaps. The sediments of these two lithological units were deposited in an outer and outer to middle ramp setting, respectively. Furthermore, they do not exhibit any major recognizable changes in sedimentation rate or detectable hiatuses.

Since different sedimentation rates within lithological units are likely, all subsequent analyses were conducted separately on each unit. Spectral analyses for both GR and MS were performed using REDFIT and Wavelet spectra (PAST version 3; http://folk.uio.no/ohammer/past/). The REDFIT settings used for each data set are shown in Figure 4. Monte Carlo simulation incorporated into REDFIT was used to further test the confidence of the detected peaks through repeated random sampling [*Schulz and Mudelsee*, 2002].

The data sets were reinterpolated using Analyseries (version 2.0.4.2; http://www.lsce.ipsl.fr/Phocea/Page/index.php?id=3). Linear interpolation was performed with double the original sample points in order to reduce aliasing of the data series. Interpolated data sets were used for both wavelet analysis as well as the used Gaussian band‐pass filters.

Band‐pass filtering of the frequencies for orbital tuning of the two lithological units was also done using Analyseries. Bandwidths used for the different Gaussian band‐pass filters on each data set were defined as 25% of the detected frequency. The resulting spectral peaks of both GR and MS were subsequently compared for each unit, respectively, to compare the spectral results of the two methodologically unrelated proxies.

Based on the calculation of average sedimentation rates, sedimentological description, and the resulting paleoenvironmental interpretation [see *Reuter et al*., 2013], only the 405 kyr eccentricity was used for subsequent orbital tuning. The 405 kyr eccentricity was selected as the primary tuning target since it represents the most stable orbital component over long time periods [e.g., *Weedon*, 2003; *Hinnov and Hilgen*, 2012].

The orbital solution used for tuning was obtained from the data incorporated in Analyseries [*Paillard et al*., 1996; *Laskar et al*., 2004]. The 405 kyr eccentricity peak of the calculated eccentricity parameter for the relevant timeframe was again filtered using Gaussian band‐pass filters of Analyseries in order to get the oscillations and amplitude of the 405 kyr heartbeat [see *Pälike et al*., 2006]. The section was then tuned by matching the filtered results of the studied proxies (GR and MS) to the calculated 405 kyr eccentricity oscillations derived from the La2004 solution [*Laskar et al*., 2004]. The initial tuning was performed on the 405 kyr eccentricity since the higher‐frequency Milankovitch cycles—although clearly present—were deemed to be too unreliable for tuning. Biostratigraphic markers as well as chemostratigraphic interpretation of the section [*Reuter et al*., 2013] were used as age control for the initial orbital tuning (Figure 6). The 405 kyr tuning allowed a subsequent tentative correlation of 100 kyr eccentricity cycles within the section (Figure 6), which allowed a comparison of the amplitude modulation of the eccentricity curve with GR and MS records of the Decontra section (supporting information Figure S3).

## 4 Results

### 4.1 Geochemical and Geophysical Measurements

**Figure 3:**
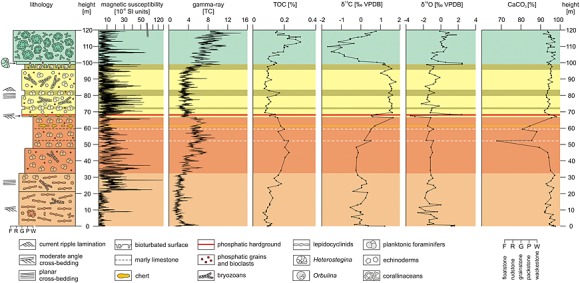
Schematic lithological profile showing sedimentary structures and lithology of the five informal lithological units of the Decontra section: *Lepidocyclina* Limestone, Cerratina cherty Limestone, Bryozoan Limestone intercalated with planktonic foraminiferal Limestones, the *Orbulina* Limestone, and the *Lithothamnium* Limestone. The units are color coded according to the scheme shown in Figure 2. Colors also indicate the units in the gathered geophysical (magnetic susceptibility and natural gamma radiation), geochemical (organic carbon and calcium carbonate contents), and stable oxygen and carbon (δ^13^C and δ^18^O) isotope records [after *Reuter et al*., 2013].

While the recorded gamma ray (GR) and magnetic susceptibility (MS) values are relatively low throughout the section, they show clear patterns (Figure 3). MS shows an overall trend of decrease in the Cerratina cherty Limestone. In contrast, the MS record in the Bryozoan Limestone shows greater variability. Similarly, an overall shift from on average higher to lower GR values coincides with the change in lithology from the Cerratina cherty Limestone to the Bryozoan Limestone.

Calcium carbonate content of the section typically varies between 90% and 95% in the Bryozoan Limestone as well as the lower part of the Cerratina cherty Limestone. Marked decreases (down to ~70%) occur in the upper part of the Cerratina cherty Limestone, which coincide with recorded occurrences of “marl layers” described in the literature [*Reuter et al*., 2013]. However, the results of the X‐ray diffraction show that the layers do not contain recognizable amounts of clay but rather significant amounts of quartz and clinoptilolite, a silica‐rich authigenic zeolite mineral (see supporting information Figures S1 and S2). Clinoptilolite often occurs in Miocene carbonate sediments in the presence of high amounts of silica [*Stonecipher*, 1976; *Nähr et al*., 1998].

Conversely, total organic carbon (TOC) is quite low throughout the section. Overall, the recorded trends of TOC seem to be directly related to lithology within the section. Average values for the Cerratina cherty Limestone are reported above ~0.2%, while the Bryozoan Limestone consistently shows values ranging from ~0.1 to ~0.15% TOC (Figure 3). The positive correlation between overall trends in TOC and GR indicates a link between the two records [*Reuter et al*., 2013].

### 4.2 Spectral Analysis

REDFIT analysis of both GR and MS measurements resulted in significant peaks for both studied units, which are summarized in Table 2. Converting periodicities in meters using sedimentation rate estimates derived from chemostratigraphic correlations [after *Reuter et al*., 2013], results in time estimates for the significant peaks, allowing a comparison with known orbital cycles.

**Table 2:** Results of the REDFIT Analyes and Calculated Periodicities in Thickness and Time^a^

	Frequency	Confidence Interval AR1(MoCA)	Periodicity (m)	Periodicity (kyr; Estimated Sedimentation Rate)	Tuned to 405 kyr Eccentricity
*Cerratina Cherty Limestone Sedimentation Rates After Reuter et al*. [2013]					
MS	0.28817	>95%	3.47	598.31	615.61
	0.43802	>90%	2.28	393.62	405.00
	1.8673	>99%	0.54	92.33	95.00
	4.4955	>99%	0.22	38.35	39.46
	5.2908	>99%	0.19	32.59	33.53
GR	0.11549	>90%	8.66	1492.89	1536.06
	0.21654	>90%	4.62	796.22	819.25
	0.43551	>90%	2.30	395.89	407.34
*Bryozoan Limestone Sedimentation Rates After Reuter et al*. [2013]					
MS	0.34036	>99%	2.94	419.72	405.00
	1.0454	>80%	0.96	136.65	131.86
	3.5003	>95%	0.29	40.81	39.38
GR	0.3397	>99%	2.94	420.54	405.79
	0.53382	>95%	1.87	267.61	258.23
	1.4559	>80%	0.69	98.12	94.68
	2.2485	<95%	0.44	63.53	61.31
	2.5235	>95%	0.40	56.61	54.63
	2.861	~95%	0.35	49.93	48.18
	3.0088	>90%	0.33	47.48	45.81

a Table 2 shows the frequencies as well as the AR1 Monte Carlo‐corrected confidence interval of the peaks detected in the REDFIT analysis. Frequencies were converted into cyclicities in meters and transformed into time‐based values using the sedimentation rate estimates derived from the stratigraphic model of *Reuter et al*. [2013]. The 405 kyr long eccentricity was then used to tune the detected periodicities of the Decontra section, with the results shown in the last column.

Calculating the sedimentation rates for both the Bryozoan and Cerratina cherty Limestones in this manner results in a good fit of the selected periodicities with the long (~405 kyr) and short (~100 kyr) eccentricity, as well as obliquity (~41 kyr). Sedimentation rates were then adjusted to improve the fit with the reported periodicities of known orbital cycles, resulting in the present age model (Table 2). Supporting information Figure S3 shows sedimentation rate estimates both units using the tentative tuning to the 100 kyr eccentricity solution of *Laskar et al*. [2004] performed in Analyseries.

**Figure 4:**
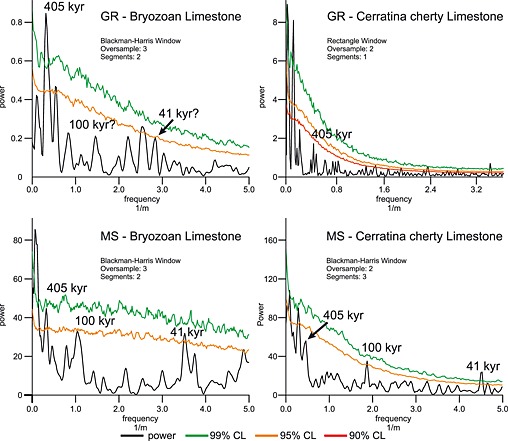
REDFIT power spectra for the two investigated lithological units of the Decontra section. Analyses for magnetic susceptibility (MS) and natural gamma radiation (GR) were performed separately for each unit. The *x* axis shows the frequency, which is the reciprocal value of the periodicity in meters. Frequencies in meters corresponding to the long and short eccentricity as well as the obliquity parameters in our current age model were labeled with periodicities in kyr.

Significant spectral peaks that fit orbital cyclicities using the estimated sedimentation rates for the Cerratina cherty Limestone are recorded at periodicities of 0.22 m, 0.54 m, and 2.28 m, respectively, with significances well above, or close to, the 95% AR1 Monte Carlo tested confidence interval (Figure 4). The gamma ray signal for the Cerratina cherty Limestone shows less significant peaks, although a peak with ~90% confidence is clearly visible at a periodicity of 2.30 m (Figure 4 and Table. 2). The lower significances for the peaks in the GR signal are most likely related to aliasing caused by the lower measurement rates related to poorer outcrop conditions and bioturbation occurring in the Cerratina cherty Limestone.

Similarly, for the Bryozoan Limestone, significant periodicities occur in the MS record at 0.29 m, 0.96 m, and 2.94 m. GR shows a strong peak at 2.94 m, but similar to the Cerratina cherty Limestone higher‐frequency peaks (0.69 m and 0.35 m) are not well reflected in the GR record. The absence of higher‐frequency peaks can be explained by the lower resolution of the GR measurements compared to that of the MS record.

**Figure 5:**
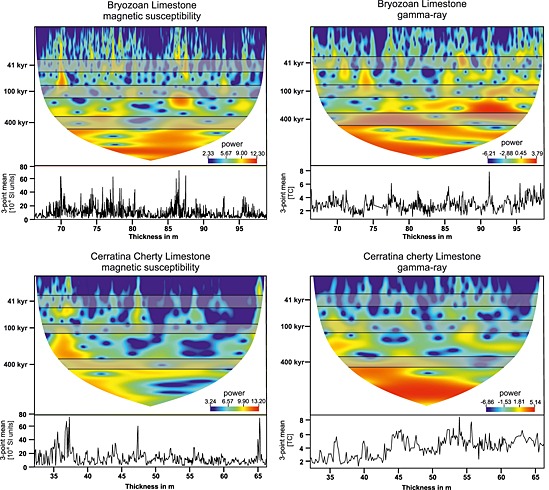
Wavelet spectra and their corresponding data sets (three‐point mean) of magnetic susceptibility and natural gamma radiation for the two investigated lithological units of the Decontra section. Gray bars indicate the relevant orbital components of long (405 kyr) and short (100 kyr) eccentricity as well as (41 kyr) obliquity, at the position of their corresponding frequencies in the wavelets. Color bar at the bottom right of each wavelet shows the relative power at different thicknesses and frequencies.

Wavelet spectra calculated using the reinterpolated data sets support the detected periodicities in GR and MS in both Cerratina cherty Limestone and Bryozoan Limestone. Additionally, the resolution of the wavelet spectra appears to be better for higher (shorter) frequencies (periodicities) for the GR signal, which in turn allows a better identification of the 100 kyr and 41 kyr peak in the Bryozoan Limestone (Figure 5). Notable is also a frequency shift in the wavelet spectrum of the GR signal in the upper part of the Bryozoan Limestone that does not occur in the spectrum of the MS signal. This discrepancy in the two records seems to be a reflection of changes affecting one proxy but not the other, rather than a significant change in sedimentation rate, which would affect both proxies in a similar fashion. For instance a shift in the Uranium fixation at the sediment‐water interface may have occurred after the end of the Monterey event that was not reflected in the activity of magnetotactic bacteria (see sections 5.1 and 5.2 for a discussion of the underlying processes governing both mechanisms).

Filters of the assumed 405 kyr periodicity were examined for shifts in amplitude in GR and MS for both studied units, respectively. Amplitudes for the MS in the Cerratina cherty Limestone are strong in the lower part of the unit. The middle part is characterized by a slow decrease in amplitude, before an increase can be observed right at the boundary to the Bryozoan Limestone. Amplitudes for GR broadly reflect the trends of MS but are generally lower and less pronounced, reflecting the low‐significance 405 kyr peak recorded in the REDFIT power spectrum of GR.

**Figure 6:**
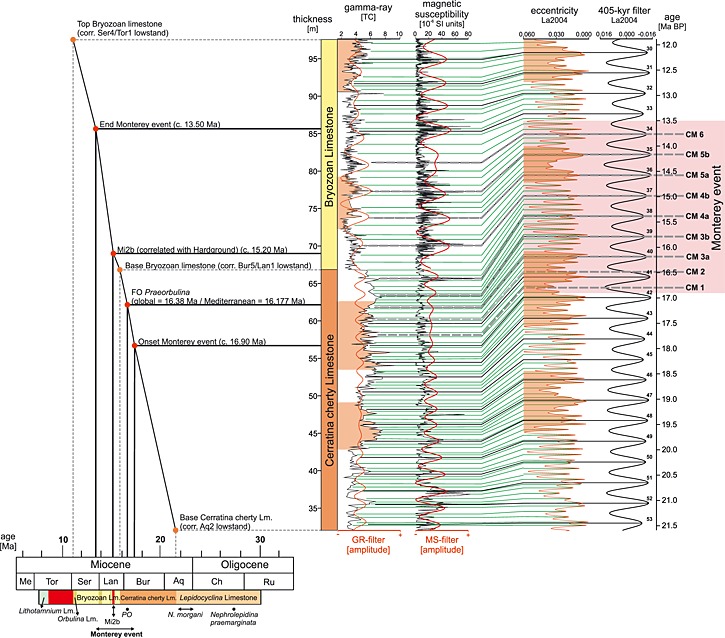
Orbital tuning of magnetic susceptibility and natural gamma radiation for the Cerratina cherty Limestone and the Bryozoan Limestone. GR and MS data are plotted in the depth domain with important tie points of the existing age model [*Reuter et al*., 2013] shown on the right‐hand side. Black lines indicate chemostratigraphic and biostratigraphic tie points, while the dashed grey lines correspond to sequence stratigraphic tie points. Both GR and MS data sets are overlain with the filtered frequencies (red curves) corresponding to the 405 kyr long eccentricity detected by REDFIT analysis (see Table 2 for details). Black correlation lines indicate the initial tuning of these filters to the filtered 405 kyr eccentricity cycle obtained from La2004 orbital solution [*Laskar et al*., 2004]. Numbers on the right of the 405 kyr filter denote the number of the individual 405 kyr cycle [*Wade and Pälike*, 2004; *Hinnov and Hilgen*, 2012]. Green lines indicate the 100 kyr fine tuning to the La2004 eccentricity solution. Orange areas highlight significant amplitude minima in eccentricity that can be correlated to long‐term maxima in the GR data of the Decontra section. The Monterey event is shown as a light pink area with the occurrence of the carbon isotope maxima (CM‐events) CM1 to CM6 shown as dashed grey lines (timing after *Holbourn et al*. [2007]) and show the correlation of the CM‐events with maxima in the GR record.

**Figure 7:**
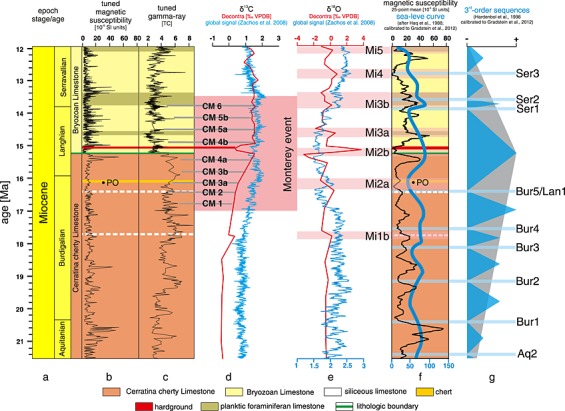
Graphic shows the tuned interval (Cerratina cherty Limestone and Bryozoan Limestone) of the Decontra section with all relevant data sets and its correlation with the global chronostratigraphy after *Gradstein et al*. [2012]: (a) Global chronostratigraphy for the tuned interval, with ages in Ma before present; (b) tuned magnetic susceptibility (MS) and (c) tuned gamma ray (GR) data of the Decontra section, colors represent the major lithologies (see Figure 2); (d) overlay of the δ^13^C record of the Decontra section (red) and the global carbon isotope stack (blue) of *Zachos et al*. [2008]; grey bars indicate the correlations of CM1 through CM6, and pink box indicates the extent of the Monterey Excursion; (e) δ^18^O data of the Decontra section (red) and its correlation with the global oxygen isotope stack (blue) of *Zachos et al*. [2008]; pink bars show the relative position of the Mi‐events and their correlation with marked changes in the lithology of the section (as seen in Figure 7f); (f) correlation of the 25‐point mean of the MS record and the global sea level curve of *Haq et al*. [1988]; light blue lines mark the correlation of minima in the Decontra MS record with the third‐order sequences of *Hardenbol et al*. [1998]. Both sea level curve and sequences are calibrated to the GTS2012 [*Gradstein et al*., 2012].

Within the Bryozoan Limestone the 405 kyr filter of MS shows constantly strong amplitudes, except for a single peak, that records a marked amplitude minimum in the middle part of the section. This amplitude minimum correlates directly with a planktonic foraminifera‐dominated Limestone that occurs in the middle part of the Bryozoan Limestone (Figures 6 and 7). The 405 kyr GR filter shows similarly strong amplitudes in the lower part of the Bryozoan Limestone. In the middle part of the section, these amplitudes, however, collapse and continue to be low and erratic until the end of the unit. Again, this marked drop in amplitudes for the GR filter coincides with a planktonic foraminifera‐dominated Limestone (Figures 6 and 7).

## 5 Discussion

### 5.1 The Relationship Between Organic Carbon, Uranium, and Gamma Radiation

*Reuter et al*. [2013] interpreted the positive correlation between TOC and GR, based on the assumption that dissolved uranium is predominantly precipitated on organic particles under reducing conditions, by decomposition of organic matter close to the sea floor [e.g., *McManus et al*., 2005]. This assumption is further supported by the low‐siliciclastic content and, thus, low content of potassium‐bearing mineral phases in the sediment [*Reuter et al*., 2013]. The later work, however, only concentrated on the general relationship between hydrological conditions, sedimentary setting, and lithology that likely influenced the larger trends observed in both TOC and gamma radiation. It is certainly true that the strong correlation between grain size and winnowing of the sediment is responsible for the large‐scale trends in both TOC and GR in the section. This, however, does not explain the distinct internal (small‐scale) variability within the otherwise more or less stable conditions that persisted throughout the deposition of the different lithological units.

Recent studies underscored the strong relationship between uranium precipitation, organic carbon rain, and prevailing redox conditions [e.g., *McManus et al*., 2005]. Authigenic uranium uptake by the sediment was found to be largely independent of possible subsequent oxidation and dissolution of organic matter [*McManus et al*., 2005]. This makes uranium an accurate proxy for the relative amount of organic carbon transported to the ocean floor [*Chase et al*., 2001; *Anderson et al*., 2002; *McManus et al*., 2005] and, more importantly, as a proxy for net‐primary productivity [*McManus et al*., 2005]. Uranium bound in a stable mineral phase is also very stable with regard to subsequent diagenetic processes [*McManus et al*., 2005].

Accepting these hypotheses, it can be assumed that higher‐order variations observed in the natural gamma radiation of the Decontra section are likely a direct proxy for burial rates of organic matter. While it is currently unknown in which mineral phase uranium is preserved in the Decontra section, only calcium fluorapatite or calcium carbonate seems likely options considering the known lithology of the section [*Klinkhammer and Palmer*, 1991; *Russell et al*., 1994; *Föllmi*, 1996; *Mutti and Bernoulli*, 2003; *Reuter et al*., 2013]. Uranium, however, is only incorporated into biogenic carbonates during their formation [*Veizer*, 1983; *ten Kuile and Erez*, 1987, 1988; *Russell et al*., 1994; *Dunk et al*., 2002], which only occurs in equilibrium with the ocean water [*Russell et al*., 1994]. This leaves only direct transport of uranium‐enriched particulate organic matter to the seafloor and subsequent authigenic mineralization as a likely source for the observed variations in the GR record.

Furthermore, dissolved uranium concentrations in the water column are rather unresponsive to changes caused by varying terrigenous input, since uranium is known to have a long residence time in the ocean (200–400 kyr) [*Veeh et al*., 1974; *Ku et al*., 1977; *Anderson*, 1982]. This essentially excludes varying terrigenous input as a source for the variations in the GR record. More recent studies have further demonstrated that the amount of particulate nonlithogenic uranium is the major factor controlling the net‐uranium‐concentration within marine sediments [*Zheng et al*., 2002].

### 5.2 Magnetic Susceptibility

Magnetic susceptibility in carbonates deposited on isolated carbonate platforms can result from two different processes: (1) Deposition of detrital paramagnetic and ferromagnetic particles by means of aeolian transport processes [e.g., *Ellwood et al*., 2006; *da Silva et al*., 2009; *Hladil et al*., 2010] and (2) formation of biogenic magnetite by bacterial activity in the sediment [*Karlin et al*., 1987; *Sparks et al*., 1990; *Hladil et al*., 2004; *Simmons et al*., 2004; *Kopp and Kirschvink*, 2008].

For aeolian transport of magnetic particles, variations in glaciation are suspected as major controlling factor. This is based on the assumption that higher surface area glaciation increased dust flux in the atmosphere through stronger surface winds, lowered surface humidity, and soil moisture, as well as increased desertification as a result of falling sea levels and decreases in vegetation [*Bar‐Or et al*., 2008]. Incidentally, this was considered by *Reuter et al*. [2013] as the major factor controlling magnetic susceptibility within the Decontra section.

However, studies on the magnetic properties of platform carbonates in recent and ancient sediments suggest that magnetic susceptibility in carbonates with low‐terrigenous input is predominantly caused by the activity of magnetotactic bacteria [*Aissaoui et al*., 1990; *McNeill*, 1990; *Maloof et al*., 2007; *Kopp and Kirschvink*, 2008]. These authigenic biogenic magnetites are usually formed close to the oxic‐anoxic transition zone (OATZ) within or immediately below the sediment‐water interface [*Kopp and Kirschvink*, 2008]. While the amount of organic carbon deposited in most sediments causes a decrease in oxygen content in both pore and bottom water, the primary amount of biogenic magnetite is positively correlated with the oxygen content of marine bottom waters [*Kopp and Kirschvink*, 2008]. This results in an inverse correlation of the activity of magnetotactic bacteria and organic carbon burial [*Hesse*, 1994; *Lean and McCave*, 1998; *Yamazaki and Kawahata*, 1998; *Kopp and Kirschvink*, 2008].

However, this relationship is only true when a significant amount of organic carbon is present in the first place to create an extended OATZ. In areas exhibiting habitually low organic carbon concentrations (≤0.4 wt %) the OATZ will never develop strong enough to facilitate an extensive population of magnetotactic bacteria [*Kopp and Kirschvink*, 2008].

Applying these assumptions to the Decontra section where TOC levels are generally low (ranging from 0.06 to 0.31 wt %), variations in magnetic susceptibility are most likely related to an increase in organic carbon burial during times of higher primary productivity that caused the formation of an OATZ conductive to the growth of magnetotactic bacteria. Conversely, during times of lower productivity the generally rather high water energy at the Maiella carbonate ramp—especially in the Bryozoan Limestone—inhibited the establishment of anoxic conditions in the sediment largely preventing the growth of magnetotactic bacteria.

### 5.3 Phase Relationship

The available evidence can now be used to propose a positive feedback of primary productivity on both GR and MS data within the Decontra section. It can thus be assumed that the phase relationship of the two records with orbital eccentricity should be the same as other primary productivity proxies. It is further well established that increases in primary productivity are generally associated with eccentricity minima during the Oligocene/Miocene [see *Cramer et al*., 2003; *Wade and Pälike*, 2004; *Holbourn et al*., 2007; *Mourik et al*., 2010; *Diester‐Haass et al*., 2013]. Based on this, an inverse correlation between the GR signal and the orbital 405 kyr eccentricity has been adopted for the tuning. Conversely, the major contribution of magnetotactic bacteria to the MS record and their link to organic matter decomposition can further explain why increases in the MS‐log seem to be largely in phase with maxima in the GR‐log.

The correlation of long‐term GR maxima and with pronounced amplitude minima of the eccentricity curve, as well as larger trends in the MS record with the eccentricity amplitude modulation, offer additional support for the assumed phase relationship (Figures 6 and S3 in the supporting information).

### 5.4 Orbital Tuning

Until recently the poor biostratigraphic resolution of the Decontra section was a well‐known problem, which was resolved by applying chemostratigraphy as a main correlation tool [e.g., *Mutti et al*., 1997; *Reuter et al*., 2013]. Nevertheless, the chemostratigraphic correlation of the section depends on a few important biostratigraphic tie points, which allowed the construction of a basic biochronology [*Reuter et al*., 2013]: (1) The first occurrence of *Praeorbulina*, (2) the occurrences of *Nephrolepidina praemarginata* and *N*. *morgani* [*Reuter et al*., 2013], and (3) planktonic foraminiferal data for the uppermost *Lithothamnium* Limestone [*Carnevale et al*., 2011].

The first occurrence (FO) of *Praeorbulina* is of particular importance as it constrains the age of the upper part of the Cerratina cherty Limestone. The currently accepted global first appearance datum (FAD) of *Praeorbulina scianus* occurs at 16.38 Ma [*Wade et al*., 2011], although the exact location and accuracy of the FAD is still debated [*Iaccarino et al*., 2011; *Turco et al*., 2011]. More specifically, the evolutionary timing of various morphotypes of the *Praeorbulina* lineage is still highly debated. Nevertheless, the appearance of *Globigerinoides sicanus* with a morphotype showing a near‐spherical outline can be dated to 16.177 Ma in the Mediterranean, which significantly postdates the FO of *Globigerinoides sicanus* (in a broad sense) in the northern Atlantic, which occurs at 16.844 Ma [*Iaccarino et al*., 2011]. Since the reported occurrence of the *Praeorbulina* lineage in the northern Atlantic significantly predates the global datum, an occurrence of older specimen cannot be excluded in the Mediterranean. However, all currently available data support a maximum age of 16.177 Ma for the first occurrence of *Praeorbulina* in the Mediterranean. Based on the currently available data, this datum also needs to be assumed as correct for the Decontra section (Figure 6).

Nevertheless, considering the uncertainties present the record of *Praeorbulina*, the FO of the taxon only offers a broadly constrained tie point by itself. Combining this loose tie point with constraints provided by chemostratigraphy allows an independent confirmation. Particularly, the correlation of the marked carbon isotope excursion correlated with the Monterey event, which lasted from ~16.9 to 13.5 Ma, would be shifted to an age of ~17.7 to ~14.3 Ma, if the maximum reported age (16.844 Ma) of the FO of *Praeorbulina* is used for the Decontra section. Using the onset of the Monterey event, defined by chemostratigraphic data, as an additional tie point, thus furthermore constrains the FO of *Praeorbulina* in the section roughly between the currently accepted local Mediterranean FO (16.177 Ma) and the proposed global FAD of *Praeorbulina sicana* (16.38 Ma) (Figure 6).

Similarly, the occurrence of *N*. *morgani* in the uppermost *Lepidocyclina* Limestone constrains the age of the base of the Cerratina cherty Limestone to the late Chattian to Aquitanian (upper SBZ23 to lower SBZ25) [*Reuter et al*., 2013]. These few age dates give a robust support for the correlation of the δ^18^O and δ^13^C records to the respective global records of *Zachos et al*. [2001b] by *Reuter et al*. [2013].

Based on this biostratigraphic and chemostratigraphic frameworks, we are now able to use the cyclic variations detected in the MS and GR record to further tune the section to the long (405 kyr) eccentricity parameter. For this approach we used assumptions resulting from the preceding discussion: (1) The total age range of the Cerratina cherty Limestone is known to be from middle Aquitanian to late Burdigalian/early Langhian (Figure 2) [*Reuter et al*., 2013] allowing rough estimates of likely sedimentation rates for this unit. (2) The age range of the Bryozoan Limestone is early Langhian to late Serravallian, based on chemostratigraphic correlations (Figure 2) [*Reuter et al*., 2013]. (3) The horizon with the FO of *Praeorbulina* cannot be older than 16.38 Ma. (4) The FO of *Praeorbulina* in the Decontra section is likely not the FAD, making this horizon likely younger than the maximum 16.177 Ma (16.38 Ma?) datum. (5) Cyclic variations in both the GR and MS record reflect variations in primary productivity that can be directly linked to changes in the orbital parameters. (6) Primary productivity maxima are correlated with minima in the long eccentricity cycle. (7) Sedimentation rates stayed reasonably constant within the lithological units of the section and no significant (recognizable) hiatuses occurred within the considered interval.

Using these assumptions, we correlated the maxima in the filters of both MS and GR data following the FO of *Praeorbulina* in the upper part of the Cerratina cherty Limestone with the first 405 kyr eccentricity minimum following the 16.177 Ma Mediterranean FO of *Praeorbulina* (Cycle 40 MI‐C5Crn using the scheme of *Wade and Pälike* [2004]). This approach results in absolute ages of 21.58 to 15.24 Ma for the Cerratina cherty Limestone (Figure 6), assuming no significant changes in sedimentation rates as supported by wavelet analysis (Figure 5). The FO of *Praeorbulina* within the Decontra section thus occurs at ~16.16 Ma (Figures 6 and 7), which slightly postdates the currently accepted FO of the taxon in the Mediterranean region of 16.177 Ma [*Iaccarino et al*., 2011].

Following this initial tuning to the 405 kyr filter, it was subsequently possible to correlate the eccentricity minima to smaller peaks in the MS and, to a smaller degree, in the lower resolution GR record. The current tuning thus results from the well‐constrained correlation with the 405 kyr eccentricity cycles, especially during the Monterey event, while offering a tentative tuning to the orbital eccentricity on a 100 kyr scale (Figure 6). The tuning is further supported by the close fit of amplitude minima within the eccentricity curve to long‐term maxima occurring in the GR record (Figure 6). Particularly, the MS record also shows a close fit to the overall amplitude modulation of the eccentricity curve for the whole section (see supporting information Figure S3). This observable relationship confirms the chosen inverse phase relationship and also offers further support for the proposed age model and tuning.

Since no significant hiatus occurs between the transition from the Cerratina cherty Limestone to the Bryozoan Limestone, we correlated the minima in both filtered MS and GR with the 405 kyr eccentricity maxima of Cycle 37 (37 MI‐C5Bnr after the scheme of *Wade and Pälike* [2004]) at 15.2 Ma. As wavelet analysis supports the assumption of relatively constant sedimentation rates for the Bryozoan Limestone (Figure 5), orbital tuning now constrains the age of this unit from 15.24 to 11.92 Ma (Figure 6). Interestingly, the shift in the depositional environment coincides closely with the end of the Middle Miocene Climate Optimum (MMCO; ~17 to ~15 Ma) and the advent of the Middle Miocene Climate Transition (MMCT, ~15 to 13.8 Ma) [*Woodruff and Savin*, 1989; *Miller et al*., 1991a; *Flower and Kennett*, 1994; *Zachos et al*., 2001b, 2008]. This suggests a direct link between the beginning of the MMCT and a marked change in the depositional environment at the Maiella Platform.

### 5.5 The Monterey Carbon Isotope Excursion

The Monterey Excursion [*Vincent and Berger*, 1985] is widely recognized in the Mediterranean [*Jacobs et al*., 1996; *John et al*., 2003; *Sprovieri et al*., 2004; *Brandano et al*., 2010; *Mourik et al*., 2010; *Reuter et al*., 2013]. In the Decontra section *Reuter et al*. [2013] were able to refine the Monterey Excursion by detailed chemostratigraphy in combination with biostratigraphy. Based on their correlation the Monterey Excursion begins in the uppermost quarter of the Cerratina cherty Limestone and ends roughly in the middle of the Bryozoan Limestone.

Internal periodic variations in the δ^13^C record of the Monterey Excursion were first recognized by *Woodruff and Savin* [1991], who defined six so‐called carbon isotope maxima (CM‐events) that occurred over the span of the excursion. These CM‐events are also described in several sections in the Mediterranean [*Jacobs et al*., 1996; *John et al*., 2003; *Abels et al*., 2005; *Mourik et al*., 2011].

More recently, improvements in orbital theory and orbital tuning of Integrated Ocean Drilling Program sites allowed a detailed interpretation of the CMs and to link their occurrences to the 405 kyr eccentricity cycle [*Holbourn et al*., 2007]. This link between carbon isotope excursions and the 405 kyr eccentricity cycle was also recognized in the Mediterranean on Malta [*Mourik et al*., 2011]. Up till now, however, only pelagic sections offered the necessary stratigraphic resolution to resolve the internal patterns and orbital forcing of this long‐lasting event.

Accepting the hypothesis that natural gamma radiation is a proxy for estimating organic carbon burial rates in isolated carbonate settings implies that maxima in GR and the carbon isotope maxima during the Monterey Excursion were caused by changes of the same ecological parameters. This, in turn, would indicate that GR maxima in the Decontra section should align well with the reported CMs of the Monterey Excursion.

Unfortunately, the 405 kyr eccentricity signal is not well expressed in the GR signal of the Cerratina cherty Limestone, as result of increased aliasing caused by lower measurement rates [see *Weedon*, 2003]. However, it is much better preserved in the Bryozoan Limestone. This makes this unit a preferential target to verify a possible correlation between GR maxima and δ^13^C maxima.

Testing this hypothesis clearly shows that the observed GR maxima in the Bryozoan Limestone coincide closely with the occurrences of CMs during the time from 15 to 13.5 Ma (Figures 6 and 7). Furthermore, while the correlation in the Cerratina cherty Limestone is not nearly as good as in the Bryozoan Limestone, tentative correlations between GR maxima and CMs 1A to 4A can still be made using the tuned age model for the Decontra section (Figures 6 and 7).

### 5.6 Evidence for Major Climate Shifts and Miocene Glaciations

Analogous to the carbon isotope maxima recorded by *Woodruff and Savin* [1991], similar events were also recorded for oxygen isotope data during the Miocene. These Miocene oxygen isotope events (Mi‐1–Mi‐7) were noticed to be globally synchronous and are related to major increases in glaciation and thus global cooling [*Miller et al*., 1991a, 1991b; *Wright and Miller*, 1992; *Miller et al*., 1998; *Westerhold et al*., 2005; *John et al*., 2011]. In this study correlation and timing of the Miocene isotope events are derived from *Westerhold et al*. [2005], *John et al*. [2011], and the data sets of the GTS2012 [*Gradstein et al*., 2012].

Using the new tuning of the Decontra section, all known oxygen isotope shifts (except Mi4) can now be directly correlated with significant sedimentological features observed in the Decontra section that can be interpreted to reflect changes in local paleoenvironmental conditions during the Mi‐events:

Mi1b can be correlated with the first occurrence of cherty limestone in the Cerratina cherty Limestone, expressed by a marked decrease in carbonate content (<70%) of the section. The generally high content of siliceous fossils in the Cerratina cherty Limestone is a result of extensive marine volcanism during the rotation of the Sardinia‐Corsica block between ~22 and 15 Ma [*Gattacceca et al*., 2007; *Brandano et al*., 2010; *Reuter et al*., 2013]. Similar increase in SiO_2_ and other nutrients causing higher primary productivity are a well‐known effect of volcanic events in the modern ocean [e.g., *Uematsu et al*., 2004].

The coincidence of a cherty layer containing silica and clinoptilolite with the Mi1b event points toward an increased input of siliceous skeletal material during that time. The silica is derived from siliceous sponges and radiolarians [*Mutti et al*., 1997; *Reuter et al*., 2013]. The increase in silicate sponges and radiolarians was likely a direct effect of the cooler temperatures favoring siliceous organisms. Increased upwelling along the Maiella ramp caused by a shift in the local current system during the global cooling may also have contributed to the formation of the cherty layer.

Similarly, Mi2a seems to be expressed by the occurrence of a second cherty layer in the Cerratina cherty Limestone, followed by a layer containing spiculitic chert nodules. Thus, production of siliceous plankton and sponges again increased during Mi2a at the Maiella Platform. The layer containing spiculitic chert nodules possibly points toward increased upwelling‐derived primary productivity causing extensive growth of siliceous sponges during the later stage of Mi2a.

Mi2b [after *John et al*., 2011] closely coincides with the end of the MMCO at ~15 Ma and is clearly expressed in the Decontra section by the occurrence of an extensive phosphatic hardground that formed shortly above the base of the Bryozoan Limestone. Phosphogenesis is mostly caused by the export of phosphor‐rich organic material from the ocean surface [*Föllmi*, 1996]. The coincidence of the formation of this localized hardground hints at early lithification caused by the formation of a biofilm during increased nutrient fluxes on the Maiella platform [*Mutti and Bernoulli*, 2003].

The onset of Mi3a can be correlated to the first occurrence of a bed of planktonic foraminiferan limestone in the lower quarter of the Bryozoan Limestone (Figure 3). Again, this marked change in sediment composition indicates a major shift in paleoenvironmental conditions during Mi3a that caused a shift from a bryozoan fragment dominated depositional regime to a more planktonic foraminifera‐dominated sediment. A possible explanation for this shift toward higher plankton productivity at the ocean surface may be the increased upwelling‐induced nutrient availability [*Thiede*, 1975; *Sautter and Thunell*, 1991; *Curry et al*., 1992; *Little et al*., 1997; *Vecsei and Sanders*, 1997; *Eguchi et al*., 1999, 2003]. Planktonic foraminiferal limestones are thus likely linked to major oceanographic changes in the Mediterranean, which reduced production of benthic carbonates by bryozoans and echinoderms and thus a decrease in the highstand shedding. This effect is likely related to the negative effect which decreasing sea levels had on carbonate production on the Maiella platform, by reducing the deposition of benthos‐derived carbonates on the ramp. Additionally, changing climate conditions may have also caused the onset of increased upwelling conditions during the Mi‐events that increased primary productivity and thus the deposition of planktonic microfossils. These changes are likely related to the changes in the general current patterns in the Mediterranean caused by an increased meridional temperature gradient [*Flower and Kennett*, 1994; *Mutti and Bernoulli*, 2003].

Conversely, the positive isotope shift of Mi3b is also expressed as a 3 m thick planktonic foraminifera‐dominated limestone and was likely caused by similar underlying mechanisms to Mi3a. Mi3b is concurrent with the middle Miocene expansion of the East Antarctic Ice Sheet and triggered the major cooling step that marked the end of the Monterey Excursion and subsequent shift into an icehouse climate in the late Miocene. Mi3b also represents the main event of the MMCT [*Miller et al*., 1991b; *Zachos et al*., 2001b, 2008; *Shevenell et al*., 2004, 2008; *Tian et al*., 2009, 2013]. This marked climatic event can also be correlated with a breakdown in the amplitude of the 405 kyr magnetic susceptibility cycle (Figure 6), further hinting at the strong ecological impact of the Mi3b event at the Maiella platform. Additionally, the amplitude minimum is likely the effect of a combined minimum in obliquity and eccentricity modulation that occurred at this time [*Laskar et al*., 2004; *Shevenell et al*., 2004]. Considering the thickness of this planktonic foraminiferal Limestone coinciding with Mi3b/CM6, a strong long‐lasting change in climate and hydrological conditions can be inferred for the Maiella platform. Similar to Mi3a increased upwelling intensity likely caused an increased primary production in the photic zone, while the benthic carbonate factory that otherwise dominated the Bryozoan Limestone suffered from a major setback during this interval. While the expansion of the Antarctic ice sheet during both Mi3a and Mi3b had a major impact on the climatic conditions, the main expression of these events at the Maiella platform were not changes in temperature, but rather changes in hydrological conditions.

Mi4 does not have a clear representation in the Decontra section. Since it represents only a minor step in a major cooling trend that started with Mi3b, it is possible that this more gradual increase in glaciation did not have a profound and immediate impact on the depositional environment of the Decontra section, allowing the bryozoan dominated carbonate ramp sediments to persist.

Mi5a [*John et al*., 2011], in contrast, is again clearly expressed at the top of the Bryozoan Limestone as another planktonic foraminiferal packstone. Considering the pacing and range of Mi5a the beginning of the *Orbulina* Limestone coincides with the onset of the associated δ^18^O event. The *Orbulina* Limestone, composed of planktonic foraminifers containing *Orbulina* spp., marks the end of the Bryozoan Limestone and also closely coincides with the third‐order lowstand of Tor1 of *Haq et al*. [1988] calibrated to *Gradstein et al*. [2012]. This major sea level lowstand is expressed as a phase of nondepositon, which caused the formation of a hardground surface [*Mutti et al*., 1997; *Reuter et al*., 2013].

In summary, nearly all Mi‐events (except Mi4) are either expressed as plankton‐rich (Mi3a, Mi3b, and Mi5), siliceous (Mi1b and Mi2), or phosphatic (Mi2b) horizons in the Decontra section. Siliceous horizons only occur in die Cerratina cherty Limestone deposited during the time of the Sardinia‐Corsica block rotation (~22–15 Ma.) [*Gattacceca et al*., 2007; *Brandano et al*., 2010] and reflect high concentrations of silica released by marine volcanism that occurred during that time, in combination with increased activity of siliceous organisms during cooler climates. The bryozoan‐ and echinoderm‐dominated carbonates of the Bryozoan Limestone generally indicate high nutrient levels [*Mutti and Bernoulli*, 2003; *Pomar et al*., 2004]. Particularly high nutrient levels during glaciations led to either the formation of phosphatic hardgrounds (Mi2b), the formation of extensive biofilms [*Mutti and Bernoulli*, 2003], or an increase in planktonic foraminifers compared to benthic bryozoan skeletal material (Mi3a, Mi3b, and Mi5). These can be explained by increased topographical upwelling at the Maiella ramp during Mi‐events that increased productivity in the photic zone. The upwelling was caused by increased current circulation in the Mediterranean, which was in turn was caused by the higher meridional temperature gradient during Mi‐events [*Flower and Kennett*, 1994; *Mutti and Bernoulli*, 2003]. Additionally, increases in polar ice may have resulted in a waning of highstand shedding on the Maiella ramp that persisted during the deposition of the Bryozoan Limestone.

Although the oxygen isotope record of the Decontra section is at least partly altered by diagenesis, general patterns related to changes in local paleoceanographic conditions are still recognizable [*Reuter et al*., 2013]. Until now, the comparatively low resolution of the bulk δ^18^O record precluded a more detailed correlation with the global oxygen isotope records.

Utilizing the new tuning for the Cerratina cherty Limestone and the Bryozoan Limestone reveals similarities in the trends observed in the bulk δ^18^O record of the Decontra section and the global oxygen isotope stack of *Zachos et al*. [2008] (Figure 7). Based on this, it is possible to correlate major trends in the global isotope record with the isotope record of the Decontra section (Figure 7). While some uncertainties still occur in the correlation, it is nevertheless possible to roughly relate major δ^18^O excursions (Mi1b to Mi5a) with positive excursions of the Decontra oxygen isotope record. Especially Mi4, which is not expressed in the lithological record, can be correlated with an excursion in the Decontra δ^18^O record (Figure 7). The comparably low resolution of the Decontra isotope record, however, unavoidably leads to considerable aliasing. While overall trends still appear well correlated, this interpretation should still be read with this caveat in mind.

Using the current age model, the prominent peak at the beginning of the Bryozoan Limestone that correlates to a hardground can thus be directly related to the termination of the MMCO and subsequently Mi2a. It was similarly interpreted by *Reuter et al*. [2013] to reflect increased upwelling in the Decontra section at the onset of the MMCT.

### 5.7 Correlation of Magnetic Susceptibility With Global Sea Level Changes

The newly available tuning also allows a correlation of smoothed maxima in the MS signal with all third‐order highstands of *Hardenbol et al*. [1998] after calibration to the new geochronology of *Gradstein et al*. [2012]. Additionally, long‐term trends in the maxima seem to follow the global sea level curve of *Haq et al*. [1988] recalibrated to *Gradstein et al*. [2012], making all third‐order cycles that occurred during that time clearly identifiable in the magnetic susceptibility record.

This correlation of sea level changes and magnetic susceptibility appears to be an expression of changes in the production of single domain magnetite by magnetotactic bacteria. This was most likely caused by variations in the intensity of the oxic‐anoxic transition zone between third‐order highstands and lowstands (see section 5.2 for comparison), as sea level rises seem to be often associated with the formation of extensive anoxia in the ocean [e.g., *Lyons et al*., 2003]. Based on this at least a decrease in oxygen levels, caused by a reduction of water energy during sea level highstands, seems likely for the Maiella carbonate ramp. The relative decrease in oxygen, in turn, facilitated higher activity of magnetotactic bacteria. This caused higher production of biogenic magnetite, which is reflected as marked positive excursions in long‐term trends of magnetic susceptibility in the Decontra section. Consequently, relative minima in the long‐term MS trends can be directly correlated to sea level lowstands.

Considering marked changes in lithology with regard to sea level changes using the orbitally tuned age model further shows that the change from the hemipelagic Cerratina cherty Limestone to the benthic skeletal carbonate dominated Bryozoan Limestone coincides with a marked highstand at 15.2 Ma. This can be explained by the fact that contrary to siliciclastic ramps, carbonatic systems favor production and deposition of benthic carbonate during higher sea levels, caused by an increased production of carbonate in shallower waters [*Vecsei and Sanders*, 1997].

Interestingly, most sea level lowstands seem to also coincide with recognizable changes in lithology within the units. For instance, two prominent cherty layers containing zeolite at ~52 m and ~60 m, and similarly, in the Bryozoan Limestone, the beginning of a ~3 m thick planktonic foraminiferal Limestone bed at ~80 m. Also, the *Orbulina* Limestone seems to coincide with a marked sea level lowstand. This correlation, however, was likely caused by the co‐occurrence of major sea level changes and the Mi‐events, which triggered major changes in the hydrological conditions and nutrient availability at the Maiella platform ramp. Falling sea levels furthermore likely hampered highstand shedding [see *Schlager et al*., 1994] of the Maiella platform, which is the underlying cause of the formation of the Bryozoan Limestone. However, while these sea level changes had major effects on the carbonate production on the platform, they did not significantly affect the depositional regime of Cerratina cherty Limestone and the Bryozoan Limestone, as their outer to middle ramp setting was too deep to be affected by relative sea level changes in the range of tenths of meters (see Figure 7).

The shift of the depositional regime from the more pelagic Cerratina cherty Limestone to the Bryozoan Limestone dominated by skeletal fragments derived from benthic organism is thus likely a cause of the long‐term sea level highstand after the Bur5/Lan1 lowstand (Figure 7).

## 6 Conclusions

The presented results were obtained from a reevaluation of the data sets from the Decontra section (Maiella, Central Italy). We applied a new integrated stratigraphy using cyclostratigraphy and eventstratigraphy in combination with an already established chemostratigraphic and biostratigraphic framework in order to significantly improve the stratigraphic resolution. Average accuracy of the new stratigraphic framework is estimated to be well below 200 kyr.

The results of the performed spectral analyses were used to orbitally tune a part of the section in order to significantly improve on the resolution of the biostratigraphy and chemostratigraphy established by *Reuter et al*. [2013]. Sedimentation rate estimates as well as a comparison of the spectral relationships of known orbital parameters and found cyclicities allowed the identification of both the long (~405 kyr) and short (~100 kyr) eccentricity, as well as obliquity (~41 kyr). Careful examination of available biostratigraphic markers (FO *Praeorbulina*), carbon isotope stratigraphy, and also the processes involved in the formation of the GR and MS signals allowed the tuning of the section to filtered long (405 kyr) eccentricity using the orbital solution La2004 [*Laskar et al*., 2004].

The results of this first tuning were confirmed using event‐based (Mi‐ and CM‐events) stratigraphy. This integrated approach resulted in an age of 21.58 to 15.24 Ma for the Cerratina cherty Limestone. The Bryozoan Limestone can be constrained to a total age of 15.24 to 11.92 Ma. Further fine‐tuning allowed a final tentative correlation of the record to the unfiltered La2004 eccentricity solution, resulting in the first tuned shallow marine section spanning the early to middle Miocene (from 21.58 to 11.92 Ma)

Prominent maxima in the GR‐log and the CM‐events [after *Woodruff and Savin*, 1991; *Holbourn et al*., 2007] revealed a striking correlation between the two, especially CM4b to CM6, which occur within the Bryozoan Limestone. In the Cerratina cherty Limestone only a tentative correlation can be established between GR maxima and the CM1 to CM4a events.

The new age model also allowed a reevaluation of the δ^18^O record of the Decontra section. Trends observed in the Decontra oxygen isotope curve can now be closely correlated to the global isotope record of *Zachos et al*. [2008]. Even considering the comparatively low resolution of the Decontra record the fit with the Mi1b to Mi5 events is clear. A prominent excursion at the beginning of the Bryozoan Limestone, which was also correlated to the end of the MMCO by *Reuter et al*. [2013], was confirmed to represent the beginning of the MMCT. Moreover, prominent facies changes occur synchronously with nearly all observed Mi‐events (except Mi4). Facies changes within the Bryozoan and Cerratina cherty Limestones are correlated to changes in global glaciation and therefore indicative of changes in the global climate.

Finally, in addition to the well‐preserved orbital cycles in the MS record long‐term trends further show a close correlation with third‐order sequences of *Hardenbol et al*. [1998] and the sea level curve of *Haq et al*. [1988].

## Supporting information



Readme

Table S1

Figure S1

Figure S2

Figure S3
