# Supplementary material for: Correlating carbon and oxygen isotope events in early to middle Miocene shallow marine carbonates in the Mediterranean region using orbitally tuned chemostratigraphy and lithostratigraphy
Source: Paleoceanography. 2015 Apr 13;30(4):332–52. doi: 10.1002/2014PA002716 (PMC4974900; doi:10.1002/2014PA002716)
Supplement: Supplementary file 1 — Readme [file PALO-30-332-s001.docx]

Auxiliary material for

Correlating CM- and Mi-Events in Miocene shallow marine carbonates in the Mediterranean region using orbitally tuned chemo- and lithostratigraphy

Gerald Auer, Werner E. Piller, Markus Reuter

(Institute for Earth Sciences, University of Graz NAWI Graz, 8010 Graz, Austria)

Mathias Harzhauser

(Geological-Paleontological Department, Natural History Museum Vienna, 1010 Vienna, Austria)

Introduction

This data set contains the geophysical (natural gamma radiation, magnetic susceptibility) and geochemical (organic carbon, calcite equivalent carbonate content, sulfur, as well as stable carbon and oxygen isotope) data, which were collected during the sampling campaign at the Decontra section in August 2012. The MS Excel file ds01 is subdivided into two tabs. The first one (“Data”) contains the complete dataset of the Decontra section as collected during the field campaign and subsequent lab analyses. The second tab (“Split MS and GR Datasets”) contains the split geophysical datasets of the Bryozoan and Cerratina cherty Limestone used for spectral analyses.

Additionally, two figures (fs01, fs02) containing the X-ray diffraction spectra of two samples (as referred to in the text), are provided in this auxiliary material. The final figure (fs03) presents a tentative tuning of the gamma ray and magnetic susceptibility dataset of both the Bryozoan and Cerratina cherty limestone to the 100 kyr-eccentricity solution of Laskar et al. [2004].

1. ds01.xlsx, Collection of all relevant datasets used in this study

1.1 Tab “Data”, contains the complete geochemical and geophysical dataset of the Decontra section.

1.1.1 Column group “LECO“, contains results of the LECO carbon and sulfur analysis. These data were used in figure 03.

1.1.1.1 Column “Sample“, Name of the analyzed sample.

1.1.1.2 Column “Thickness”, Position of the samples, given as the cumulative thickness in the section.

1.1.1.3 Column “TOC (%)”, Content of organic carbon in the analyzed samples, in percent.

1.1.1.4 Column “CaCO3 (%)”, Content of calcite equivalent carbonate in the analyzed samples, in percent.

1.1.1.5 Column “S (%)”, Content of sulfur in the analyzed samples, in percent.

1.1.2 Column group “magnetic susceptibility”, contains the results of magnetic susceptibility (given as 10^-6^ SI units). These data were used in figure 03 and 06.

1.1.2.1 Column “Layer”, denotes the beginning of each layer, recorded in the section.

1.1.2.2 Column “Thickness”, denotes the center positon of each measurement, as cumulative thickness for the whole section.

1.1.2.3 Column “MS”, denotes the measurement results of the handheld MS-device (written as 10^-6^ SI units).

1.1.3 Column group “natural gamma radiation”, contains the results of the field measurements of natural gamma radiation (given as total counts per second). These data were used in figure 03 and 06.

1.1.3.1 Column “Layer”, denotes the beginning of each layer, recorded in the section.

1.1.3.2 Column “Thickness”, denotes the center position of each measurement, as cumulative thickness for the whole section.

1.1.3.3. Column “GR”, denotes the measurement results of the handheld GR-device (written as total counts per Second).

1.1.4 Column group “stable isotopes”, contains the results of the stable oxygen and carbon isotope analyses in the lab. These data were used in figure 03 and 06.

1.1.4.1 Column “Sample“, Name of the analyzed sample.

1.1.4.2 Column “Thickness”, position of the samples, given as the cumulative thickness in the section.

1.1.4.3 Column “d13C”, gives the results of the isotope analyses for carbon using the standard delta notation using the VPDB standard.

1.1.4.4 Column “d18O”, gives the results of the isotope analyses for oxygen using the standard delta notation using the VPDB standard.

1.2 Tab “Split MS and GR Datasets”, split geophysical dataset of the Cerratina cherty Limestone and the Bryozoan Limestone used for spectral analyses.

1.2.1 Column group “Cerratina cherty Limestone”, contains the geophysical measurements for the lithological unit called the Cerratina cherty Limestone used for spectra analyses. These data were used for the REDFIT analysis (figure 04) and the wavelet analyses (figure 05).

1.2.1.1 Column “Thickness”, denotes the center position of each Magnetic susceptibility measurement, as cumulative thickness for the whole Cerratina cherty Limestone.

1.2.1.2 Column “MS”, denotes the measurement results of the handheld MS-device (written as 10^-6^ SI units).

1.2.1.3 Column “Thickness”, denotes the center position of each natural gamma-ray measurement, as cumulative thickness for the whole section.

1.2.1.4 Column “GR”, denotes the measurement results of the handheld GR-device (written as total counts per Second).

1.2.2 Column group “Bryozoan Limestone”, contains the geophysical measurements for the lithological unit called the Cerratina cherty Limestone used for spectra analyses. These data were used for the REDFIT analysis (figure 04) and the wavelet analyses (figure 05).

1.2.2.1 Column “Thickness”, denotes the center position of each Magnetic susceptibility measurement, as cumulative thickness for the Bryozoan Limestone.

1.2.2.2 Column “MS”, denotes the measurement results of the handheld MS-device (written as 10^-6^ SI units).

1.2.2.3 Column “Thickness”, denotes the center position of each natural gamma-ray measurement, as cumulative thickness for the whole section.

1.2.2.4 Column “GR”, denotes the measurement results of the handheld GR-device (written as total counts per Second).

2 fs01. Results of the X-ray diffraction analysis for sample DC3_19_1Ml, which represents the first ‘marly layer’ recorded in the Cerratina cherty Limestone. Measurement was performed with a Siemens D5000 X-ray diffractometer, at the University of Graz. Measurement area was defined as 4.10° to 60.0° with a step-length of 0.05°, and a step time of 2s. Red lines correspond to peaks caused by calcite, blue lines correspond to lines caused by quartz, and green lines correspond to lines caused by the presence of the zeolite mineral clinoptilolite.

3 fs02. Results of the X-ray diffraction analysis for sample DC3_21_HG_Base, which represents the base of the prominent hardground recorded in the Bryozoan Limestone. Measurement was performed with a Siemens D5000 X-ray diffractometer, at the University of Graz. Measurement area was defined as 4.10° to 60.0° with a step-length of 0.05°, and a step time of 2s. Red lines correspond to peaks caused by calcite. No other minerals were recognized in the sample. Slight peak shifts from the ideal 2-theta values are caused by the incorporation of low quantities of MgCO3 into the calcite.

4 fs03. Tentative tuning of the gamma-ray and magnetic susceptibility datasets of both the Bryozoan and Cerratina cherty limestone to the 100 kyr-eccentricity solution of Laskar et al. [2004], based on the correlation shown in Figure 6. Ages are given in kyr before present [BP]. Datasets are plotted in their outlier corrected and detrended form and overlain by the orbital solution of Laskar et al. [2004] in red to compare the amplitude modulation of the eccentricity curve with peaks in the dataset. Both gamma ray and magnetic susceptibility data are split into a green and blue part, respectively representing the portion of the data acquired in the Bryozoan Limestone and the Cerratina cherty Limestone. Sedimentation rate estimates (shown in mm/kyr) resulting from the linear correlation of peaks using the Analyseries (ver. 2.0) ‘Linage’ tool are plotted besides the two datasets. A calculated average (dashed red line) is given for each respective unit.
