# Supplementary material for: Correlating carbon and oxygen isotope events in early to middle Miocene shallow marine carbonates in the Mediterranean region using orbitally tuned chemostratigraphy and lithostratigraphy
Source: Paleoceanography. 2015 Apr 13;30(4):332–52. doi: 10.1002/2014PA002716 (PMC4974900; doi:10.1002/2014PA002716)
Supplement: Supplementary file 3 — Figure S1 [file PALO-30-332-s003.pdf]

# DC3_19_1Ml

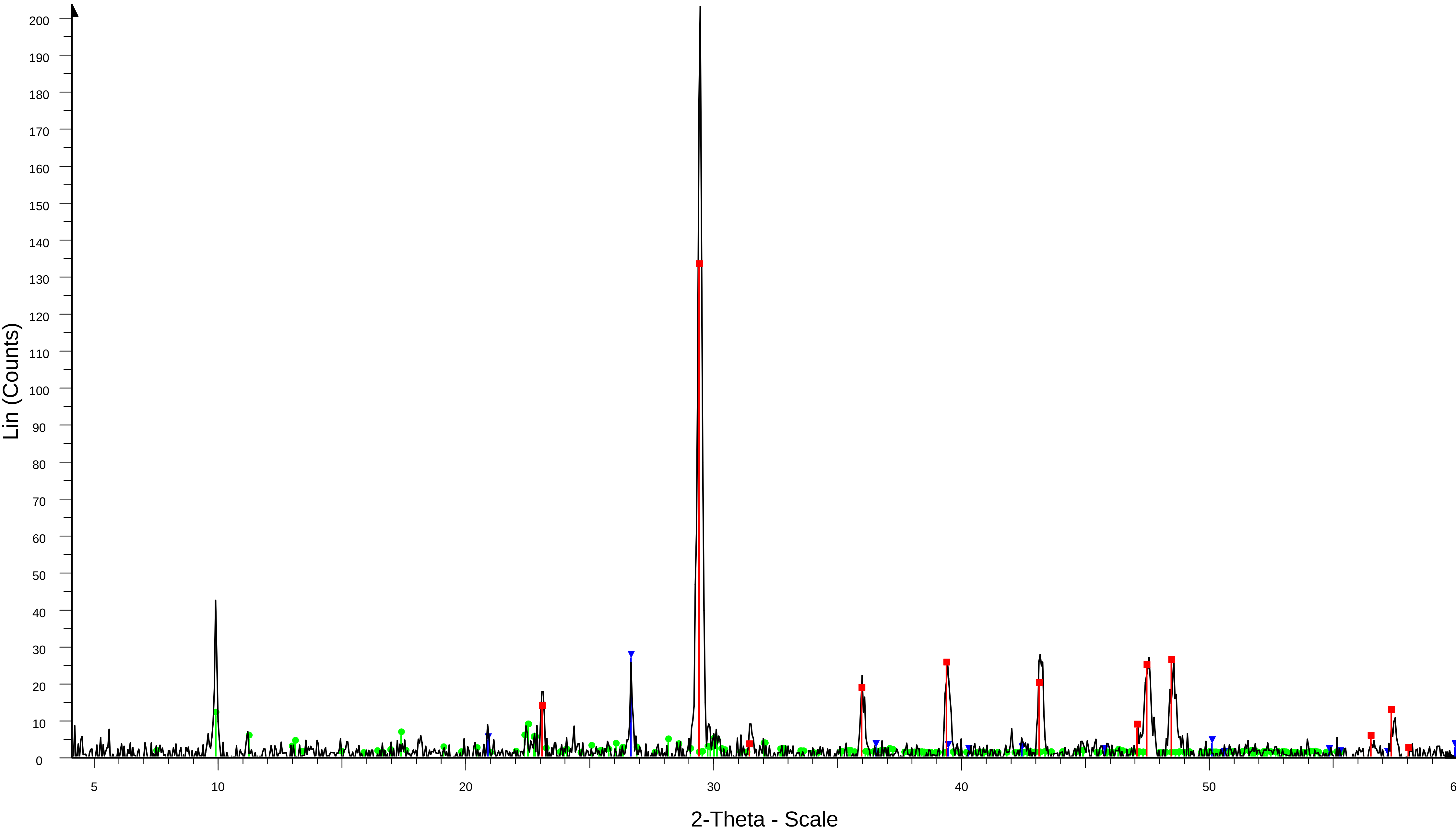


DC3_19_1Ml - File: DC3_19_1Ml.raw - Type: 2Th/Th locked - Start: 4.100 ° - End: 60.000 ° - Step: 0.050 ° - Step time: 2. s - Temp.: 25 °C (Room) - Time Started: 0 s - 2-Theta: 4.100 ° - Theta: 2.050 ° - Phi: 0.00 ° - Display plane: 1

Operations: Background 1.000,1.000 | Import

01-080-1557 (C) - Clinoptilolite - from Richardson Ranch, Oregon, USA - $Na_{1.66}K_{2.56}Ca_{1.9}(Al_{5.48}Si_{30.52}O_{72})(H_2O)_{19.16}$ - Y: 5.39 % - d x by: 1. - WL: 1.54056 - Monoclinic - a 17.63300 - b 17.94100 - c 7.40000 - alpha 90.000 -

00-046-1045 (*) - Quartz, syn - $SiO_2$ - Y: 13.12 % - d x by: 1. - WL: 1.54056 - Hexagonal - a 4.91344 - b 4.91344 - c 5.40524 - alpha 90.000 - beta 90.000 - gamma 120.000 - Primitive - P3221 (154) - 3 - 113.010 - I/Ic PDF 3.4 - S-Q

01-083-1762 (C) - Calcite - from Iceland - $Ca(CO_3)$ - Y: 65.14 % - d x by: 1. - WL: 1.54056 - Rhombo.H.axes - a 4.98960 - b 4.98960 - c 17.06100 - alpha 90.000 - beta 90.000 - gamma 120.000 - Primitive - R-3c (167) - 6 - 367.847
