# Supplementary material for: Correlating carbon and oxygen isotope events in early to middle Miocene shallow marine carbonates in the Mediterranean region using orbitally tuned chemostratigraphy and lithostratigraphy
Source: Paleoceanography. 2015 Apr 13;30(4):332–52. doi: 10.1002/2014PA002716 (PMC4974900; doi:10.1002/2014PA002716)
Supplement: Supplementary file 4 — Figure S2 [file PALO-30-332-s004.pdf]

DC3_21_HG_Base

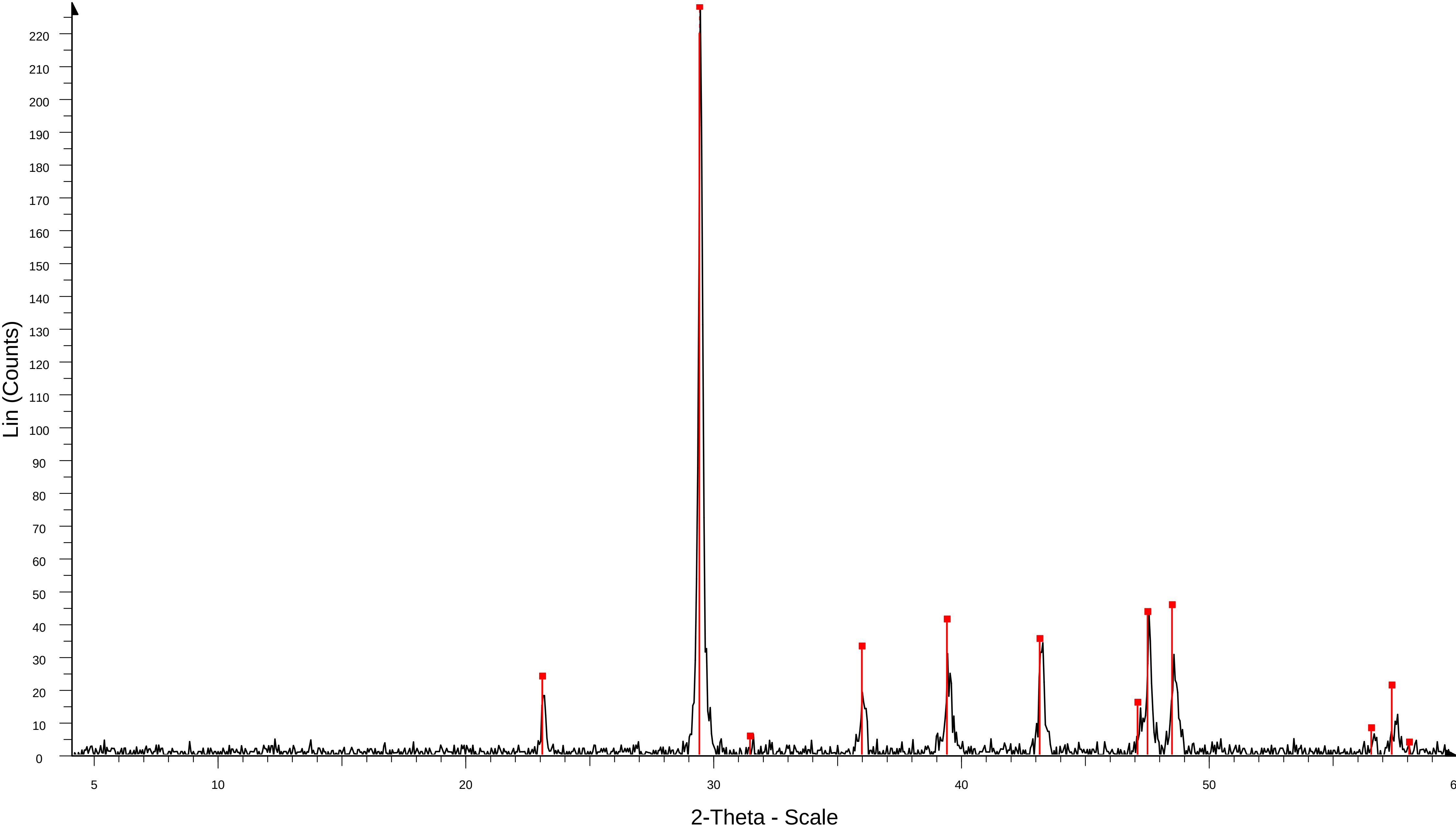

DC3_21_HG_Base - File: DC3_21_HG_Base.raw - Type: 2Th/Th locked - Start: 4.100 ° - End: 60.000 ° - Step: 0.050 ° - Step time: 2. s - Temp.: 25 °C (Room) - Time Started: 0 s - 2-Theta: 4.100 ° - Theta: 2.050 ° - Phi: 0.00 ° - Disp
Operations: Background 1.000,1.000 | Import
01-083-0578 (C) - Calcite - Ca(CO3) - Y: 100.00 % - d x by: 1. - WL: 1.54056 - Rhombo.H.axes - a 4.98870 - b 4.98870 - c 17.05290 - alpha 90.000 - beta 90.000 - gamma 120.000 - Primitive - R-3c (167) - 6 - 367.539 - I/Ic PDF 3.2
