# Supplementary figures and images for: Correlating carbon and oxygen isotope events in early to middle Miocene shallow marine carbonates in the Mediterranean region using orbitally tuned chemostratigraphy and lithostratigraphy
Source: Paleoceanography. 2015 Apr 13;30(4):332–52. doi: 10.1002/2014PA002716 (PMC4974900; doi:10.1002/2014PA002716)

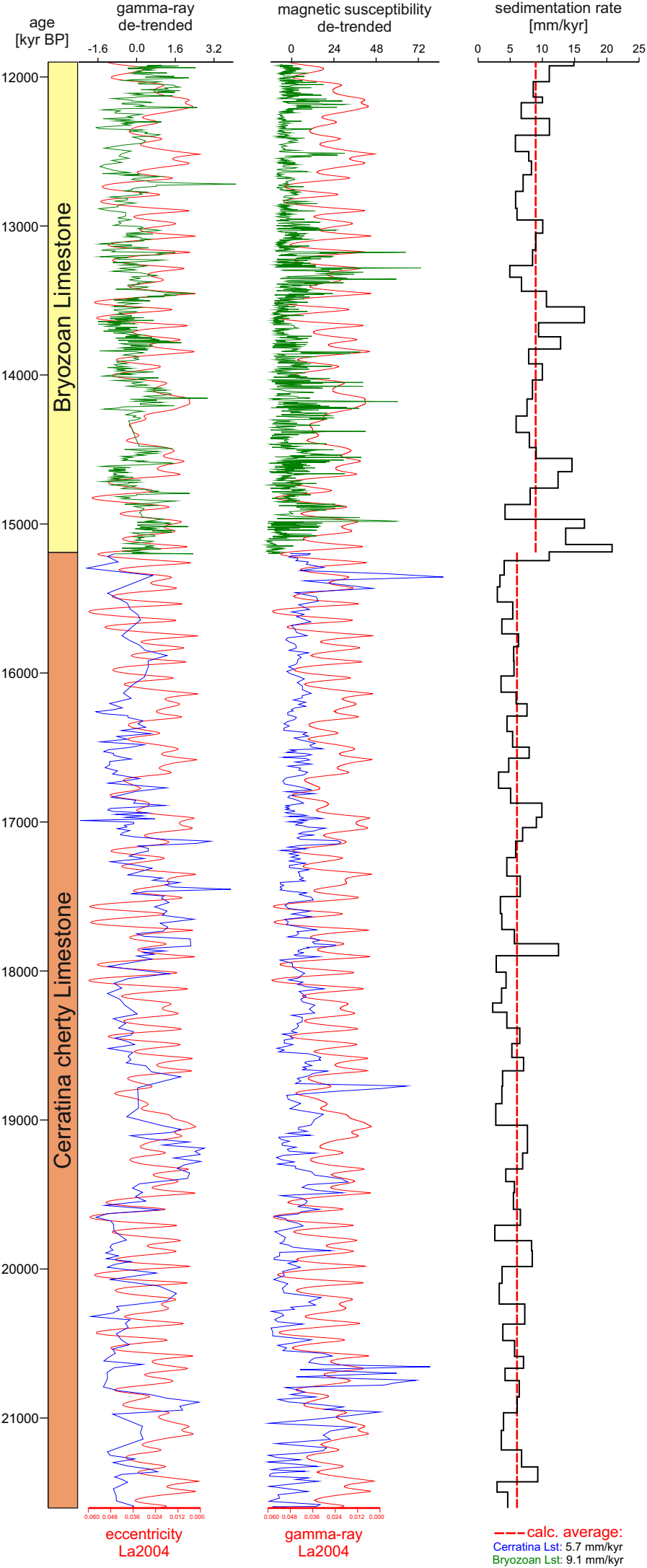

Supplement: Supplementary file 5 — Figure S3 [file PALO-30-332-s005.pdf]
